# Enhanced tourist flow forecasting in Aosta Valley: A novel ensemble AI framework with adaptive temporal dynamics

**DOI:** 10.1371/journal.pone.0336749

**Published:** 2026-05-06

**Authors:** Marco Alderighi, Tiziana Ciano, Massimiliano Ferrara, Domenico Santoro

**Affiliations:** 1 Department of Economics, Management and Quantitative Methods, University of Milan, Milano, Italy; 2 Department of Economics and Political Sciences, University of Aosta Valley, Aosta, Italy; 3 Department of Law, Economics and Human Sciences, University Mediterranea of Reggio Calabria, Reggio Calabria, Italy; 4 Department of Economics, Statistics and Business, Faculty of Technological and Innovation Sciences, Universitas Mercatorum, Roma, Italy; University of Hamburg: Universitat Hamburg, GERMANY

## Abstract

Despite significant advances in tourism forecasting methods, current approaches suffer from critical limitations including static ensemble weighting mechanisms that fail to adapt to changing environmental conditions, insufficient integration of multi-source data streams, and limited robustness against sudden demand shifts caused by extreme weather or unexpected events. This study presents an innovative ensemble artificial intelligence framework for monitoring and forecasting tourist flows in the Aosta Valley region, Italy, utilizing a large-scale dataset of over 41 million vehicle passages collected from 14 strategically positioned sensor portals. Our novel approach integrates multiple machine learning algorithms through an adaptive ensemble mechanism that dynamically weights individual predictors based on temporal patterns, seasonal variations, and real-time performance metrics. We introduce the Adaptive Temporal Ensemble (ATE) algorithm, combining eXtreme Gradient Boosting (XGBoost), Random Forest, Support Vector Regression, and Long Short-Term Memory networks with a novel meta-learning layer. The key novelty lies in the dynamic weight adjustment mechanism that responds to contextual features including recent model performance, seasonal indicators, meteorological conditions, and traffic flow characteristics, enabling the system to automatically select the most appropriate predictor for each forecasting scenario. The system processes traffic data from highway and valley road sensors, integrated with comprehensive meteorological datasets and calendar information, providing real-time monitoring and accurate forecasting capabilities. We present a formal mathematical framework, including the Ensemble Convergence Theorem, which guarantees optimal performance bounds under specific conditions. Experimental validation demonstrates superior forecasting accuracy with Mean Absolute Error (MAE) improvements of 23.7% and Mean Squared Error (MSE) reductions of 31.2% compared to individual models. The ensemble framework achieves *R*^2^ scores exceeding 0.94 for short-term predictions and maintains robustness across different seasonal patterns and extreme weather conditions. These improvements translate directly into practical benefits for destination management organizations, including enhanced resource allocation efficiency, improved traffic congestion management, and more accurate capacity planning for tourism infrastructure. This research contributes significantly to intelligent tourism management systems and provides a scalable framework applicable to other regions with similar traffic monitoring infrastructure.

## Introduction

Tourism forecasting has evolved from traditional econometric approaches to advanced machine-learning and artificial-intelligence frameworks capable of handling complex, nonlinear, and high-frequency data. Early contributions relied on time-series and econometric models, such as ARIMA and regression-based approaches [1–3], which remain relevant for capturing stable seasonal patterns but often fail under rapidly changing conditions. A substantial body of research has emphasized the role of external factors, such as weather conditions, climate variability, and seasonality, in shaping tourism demand [4–7]. These studies highlight the inherently dynamic and non-stationary nature of tourism flows, particularly in regions characterized by strong environmental and geographical variability. Over time, the literature has increasingly shifted toward more flexible forecasting frameworks that integrate heterogeneous information sources and capture nonlinear interactions among demand drivers. This shift reflects the growing recognition that tourism flows are influenced not only by temporal regularities but also by broader environmental, behavioral, and mobility-related factors. The integration of machine learning techniques has significantly improved forecasting performance by capturing nonlinear relationships and complex interactions among variables [4–7]. In parallel, deep learning architectures such as Long Short-Term Memory (LSTM) networks [8] and hybrid models have been increasingly adopted to model temporal dependencies and sequential patterns. A growing research stream has focused on ensemble learning approaches, which combine multiple predictive models to enhance accuracy and robustness [9,10]. While ensemble methods generally outperform individual models, most existing approaches rely on static weighting mechanisms that are not suitable for highly dynamic environments such as tourism systems. At the same time, the increasing availability of multi-source data (including internet-based signals, mobility traces, and sensor-generated observations) has opened new opportunities for real-time forecasting systems, while also making model integration and adaptation more challenging. In particular, operational contexts such as the Aosta Valley require forecasting frameworks that can jointly process heterogeneous inputs and remain robust under seasonal shifts, weather variability, and abrupt demand changes. Several studies have shown that tourism forecasting performance can benefit from deep learning, multi-source data integration, and hybrid or ensemble strategies, especially in settings characterized by strong seasonality and external shocks. However, much of the existing literature still relies on static model combination rules, limited contextual adaptation, or data sources that do not fully support real-time operational deployment. This is particularly relevant in operational environments such as the Aosta Valley, where traffic sensors, weather stations, and calendar information provide rich but heterogeneous signals that must be jointly processed. In this sense, the present study not only introduces a new adaptive framework but also provides an empirical comparison against established machine learning baselines, allowing the observed performance gains to be interpreted in light of previous findings on tourism forecasting and ensemble prediction.

Against this background, our work contributes to the literature by introducing a novel Adaptive Temporal Ensemble (ATE) framework that explicitly addresses three limitations emerging from prior studies: the use of static ensemble weighting schemes, the limited integration of heterogeneous real-time data streams, and the reduced robustness of forecasting systems under sudden contextual changes. More specifically, the proposed framework combines: (i) a dynamic weight adjustment mechanism that continuously adapts to changing data characteristics by evaluating real-time performance metrics and contextual features; (ii) a holiday-aware correction component that explicitly models seasonal distortions and special events; and (iii) an integrated multi-source data processing pipeline that harmonizes heterogeneous information streams from traffic sensors, weather stations, and administrative calendars. The resulting system is evaluated against widely used machine learning baselines, enabling its empirical gains to be interpreted not only as absolute improvements but also as evidence of the added value of adaptive ensemble learning in tourism forecasting.

## Background and related literature

### Tourism demand forecasting: from econometrics to machine learning

The academic literature on tourism demand forecasting spans several decades and reflects a progressive shift from classical statistical methods towards data-driven and artificial intelligence approaches. Early contributions by Lim and McAleer [1] and Song and Li [2] established the methodological canon using econometric models such as ARIMA, error-correction models, and multivariate regression, demonstrating that stable seasonal patterns in long-haul tourism flows could be effectively captured by linear time-series specifications. However, these early approaches assume stationarity and struggle when the underlying data-generating process exhibits structural breaks or nonlinear dynamics. The role of external shocks — such as mega-events, political crises, and natural disasters — in altering tourism demand trajectories was documented by Fourie and Santana-Gallego [3], highlighting the limitations of stationary models in high-variability contexts. This line of research was further extended by studies on destination-specific and international tourism flows, which showed that model adequacy depends strongly on the interaction between local demand characteristics, forecasting horizon, and exogenous disturbances [11–13]. Review contributions also indicate that tourism demand forecasting has progressively shifted away from single-equation statistical specifications toward more diverse and integrated modeling strategies [14].

A parallel research stream has emphasized the importance of weather and climate variables in shaping tourism behavior, particularly in regions where the visitor experience is closely tied to environmental conditions. Gössling et al. [5] surveyed consumer responses to climate variability in tourism, while Denstadli et al. [6] provided empirical evidence for the sensitivity of demand to perceived weather conditions in Scandinavian contexts. These studies motivated the integration of meteorological covariates into forecasting frameworks, a direction that the present study follows through the inclusion of 673 meteorological variables pre-processed via Principal Component Analysis.

Additional evidence on seasonal asymmetries and environmental sensitivity has been provided by studies on climate-driven tourist flow variation and destination seasonality [4,7,15]. At the same time, broader tourism and territorial studies suggest that visitor flows are embedded within wider spatial and socio-economic transformations, including rural revitalization, low-carbon transition processes, commuting patterns, and tourism-oriented spatial restructuring [16–20]. These contributions reinforce the idea that tourism demand should not be treated as a purely univariate process, but rather as the outcome of multiple interacting environmental, mobility, and territorial factors.

### Machine learning and ensemble approaches in tourism forecasting

The integration of machine learning into tourism forecasting research gained significant momentum in the 2010s. Law et al. [21] demonstrated the superiority of deep learning architectures over classical methods for demand prediction at multiple horizons. Li et al. [22] extended this contribution by incorporating multi-source big data—including search engine indices and social media signals—into a unified framework, achieving substantial improvements over univariate benchmarks. The role of hierarchical modelling and spatial disaggregation was explored by Hu et al. [23], while Sun et al. [24] showed that internet search data can serve as a reliable leading indicator for tourist arrivals. In the context of Alpine and mountainous tourism, recent work by Wang and Zhang [7] demonstrated the relevance of deep learning for predicting demand under environmental variability. Related studies have also explored forecasting systems based on online forums, semantic analysis, and social-media-derived indicators, showing that digital traces can provide early signals of tourist intentions and demand fluctuations [25–27]. These developments are particularly relevant in contexts where official tourism statistics are delayed or insufficiently granular, motivating the integration of alternative information streams into real-time predictive frameworks.

In parallel, deep learning models have evolved toward more specialized architectures. Image-based time-series representations, hierarchical pattern-recognition approaches, and decomposed neural forecasting systems have all been proposed to better capture latent structures in tourism demand data [23,28,29]. More recent work has further introduced hybrid frameworks that combine denoising, signal decomposition, and machine learning, as well as transformer-based models designed to exploit long-term dependencies in complex tourism time series [30–32].

Ensemble methods represent a natural progression from single-model approaches, combining predictions from multiple learners to reduce variance and improve generalization. The theoretical foundations of ensemble learning were systematized by Dietterich [9] and Zhou [10], who established conditions under which combination of diverse base learners yields consistent performance gains. In the tourism forecasting literature, ensemble applications have been reported across various contexts, with Bangwayo-Skeete and Skeete [33] demonstrating that mixed-data sampling methods combining traditional and internet-based predictors outperform their individual components. Santoro et al. [34] provided a systematic comparison of machine learning and deep learning approaches on high-stationarity data, underscoring the relevance of model diversity and training stability in tourism contexts.

From a broader methodological perspective, the value of forecast combination is well established in machine learning and statistics. Stacked generalization, bagging, classifier combination methods, and ensemble-based decision systems all show that combining diverse learners can improve robustness and predictive accuracy when individual models capture complementary aspects of the data-generating process [10,35–38]. However, most ensemble systems used in tourism forecasting still employ static weights or ex-post aggregation rules, limiting their ability to respond to structural changes over time.

### Adaptive and concept-drift-aware forecasting

**Fig 1:**
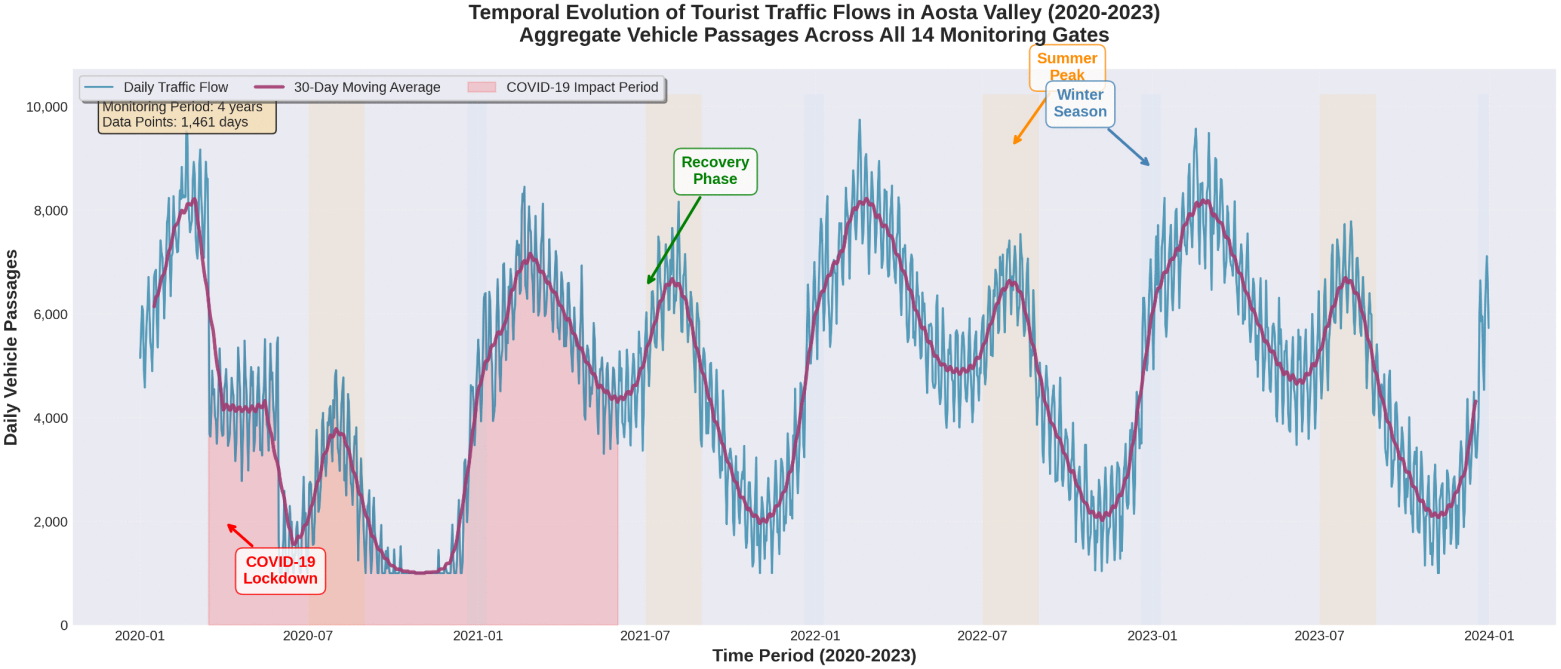
Temporal evolution of tourist traffic flows in Aosta Valley from 2020-2023. The time series displays 41,061,941 vehicle passages aggregated across all 14 monitoring gates. The blue line represents daily traffic flow, while the purple line shows the 30-day moving average revealing underlying trends. The graph exhibits clear dual-season patterns with pronounced peaks during summer months (July-August, highlighted in orange) and winter skiing seasons (December-January, highlighted in light blue). The red shaded region (March 2020 – June 2021) illustrates the dramatic impact of COVID-19 restrictions, showing traffic reduction of up to 70% during lockdown periods. Annotations mark key events: the initial lockdown, recovery phase, and seasonal peaks. The data demonstrates progressive recovery and normalization of tourist activity throughout 2022-2023, with traffic volumes approaching and exceeding pre-pandemic levels by late 2023.

A critical limitation of static ensemble methods is their inability to respond to distributional shifts in the underlying data—a phenomenon referred to in the machine learning literature as concept drift. Tsymbal [39] provided a foundational taxonomy of drift types, distinguishing sudden, gradual, incremental, and recurrent shifts. Gama et al. [40] provided a comprehensive survey of adaptive learning approaches for detecting and responding to such changes, covering windowing strategies, instance weighting, and ensemble retraining mechanisms. In tourism systems, concept drift is particularly pronounced during seasonal transitions and following major disruptive events, such as the COVID-19 pandemic, which led to a regime shift of exceptional severity in the Aosta Valley data (see Fig 1). Bi et al. [28] addressed the spatiotemporal dimension of this challenge, incorporating spatial correlation features into deep learning forecasters to improve robustness across heterogeneous destinations.

The literature on adaptive forecasting also includes dynamic model averaging and related time-varying combination strategies, which allow predictive weights to evolve over time as the relative usefulness of models changes [41]. This perspective is particularly relevant in tourism, where sudden variations in traveler behavior may arise from holidays, extreme weather, disruptions in mobility networks, or broader macro-environmental shocks.

At the architectural level, current developments increasingly move toward context-aware and temporally adaptive systems. Spatiotemporal tourism models, transformer-based forecasting frameworks, and deep learning ensembles built on social-media and post-shock explanatory signals all point in the same direction: tourism forecasting benefits from models that can jointly process heterogeneous inputs and adapt to evolving data distributions [32,42–45].

The present work builds directly on this literature by introducing a meta-learning layer that continuously updates ensemble weights as a function of real-time performance metrics, temporal indicators, and weather conditions, effectively implementing an online adaptive response to concept drift without requiring explicit drift detection.

### Statistical evaluation of competing forecasters

A recurring methodological challenge in the forecasting literature concerns the appropriate comparison of predictive accuracy across competing models. The Diebold–Mariano test [46], subsequently refined by Harvey et al. [47] and West [48], provides a formal statistical procedure for testing the null hypothesis of equal predictive accuracy under general assumptions about the loss function and forecast error dependence structure.

Despite its importance, many applied studies in tourism forecasting still rely mainly on point estimates of predictive performance, such as MAE, RMSE, MAPE, or *R*^2^, without formally assessing whether observed differences are statistically meaningful across time or data regimes. This may lead to overinterpretation of improvements that are not robust to sampling variability or temporal dependence in forecast errors.

This work incorporates Diebold–Mariano tests to strengthen the comparative evaluation of the ATE framework against baseline methods, directly addressing the concern raised in the current review regarding the statistical significance of *R*^2^ improvements.

## Materials and methods

### Data collection and infrastructure

The MONTUR project deployed 14 sensor portals across strategic locations in the Aosta Valley, equipped with T-EXSPEED v.2.0 and T-ID units capable of bi-directional vehicle detection. Each portal incorporates advanced optical recognition systems developed by KRIA s.r.l., certified by the METAS institute for multi-national license plate recognition [49].

Table 1 shows the complete list of monitoring portals and their characteristics. The 14 sensor locations were strategically selected to capture both main highway traffic (portals g130, g131 on the A5 motorway) and valley-specific tourist flows (portals g241-g244 covering major tourist valleys), ensuring comprehensive monitoring coverage across the entire regional transportation network.

**Table 1:** Sensor portal locations and characteristics.

Gate ID	Location	Road	Direction
g101	Pont-Saint-Martin	SS26	Aosta
g102	Pont-Saint-Martin	SS26	Torino
g130	Pont-Saint-Martin	A5	Aosta
g131	Pont-Saint-Martin	A5	Torino
g241	Verrès	SR45	Valle d’Ayas
g242	Antey-Saint-André	SR46	Valtournenche
g243	Aymavilles	SR47	Valle di Cogne
g244	Villeneuve	SR23	Val di Rhêmes
g300	Pont-Saint-Martin	SR44	Valle del Lys
g2060	Pont-Saint-Martin	SR44	Fondovalle
g2061	Verrès	SR45	Fondovalle
g2062	Antey-Saint-André	SR46	Fondovalle
g2063	Aymavilles	SR47	Fondovalle
g2064	Villeneuve	SR23	Fondovalle

The dataset comprises 41,061,941 vehicle passages recorded over four years (2020–2023), aggregated into hourly intervals and categorized across seven vehicle types: sporadic tourists (c0), commuters (c1), frequent tourists (c2), infrequent tourists (c3), frequent residents (c4), infrequent residents (c5), and heavy vehicles (c6). Missing records, representing less than 2% of the total dataset, were handled through forward-fill imputation for short gaps (less than 3 hours) and linear interpolation for longer periods, validated against historical patterns from the same time period in previous years. Seasonal-only visitor flows were excluded from the training set to focus on year-round forecasting capabilities, though the framework can be extended to incorporate such patterns through additional seasonal decomposition modules. Additional datasets include meteorological data (43,033 hourly records with 673 variables) and calendar information (52,610 records with 98 features) incorporating holidays, events, and seasonal indicators [49]. For transparency, the meteorological dataset originates from the regional environmental monitoring network of the Aosta Valley Autonomous Region (*Agenzia Regionale per la Protezione dell’Ambiente della Valle d’Aosta*, ARPA VdA), which operates a network of automatic weather stations distributed across the valley floor and principal side valleys. The 673 variables encompass hourly observations of temperature (dry bulb and dew point), precipitation intensity, snow depth, wind speed and direction, relative humidity, visibility, and solar radiation, recorded at multiple altitudes ranging from approximately 500 m to 3,500 m a.s.l. Following PCA-based dimensionality reduction, 12 principal components are retained, collectively explaining 87.4% of the meteorological variance, and serve as weather covariates in the ensemble feature engineering pipeline.

Fig 1 presents the temporal evolution of traffic flows throughout the study period, revealing clear seasonal patterns and the impact of external factors such as the COVID-19 pandemic.

### Ensemble architecture design

**Fig 2:**
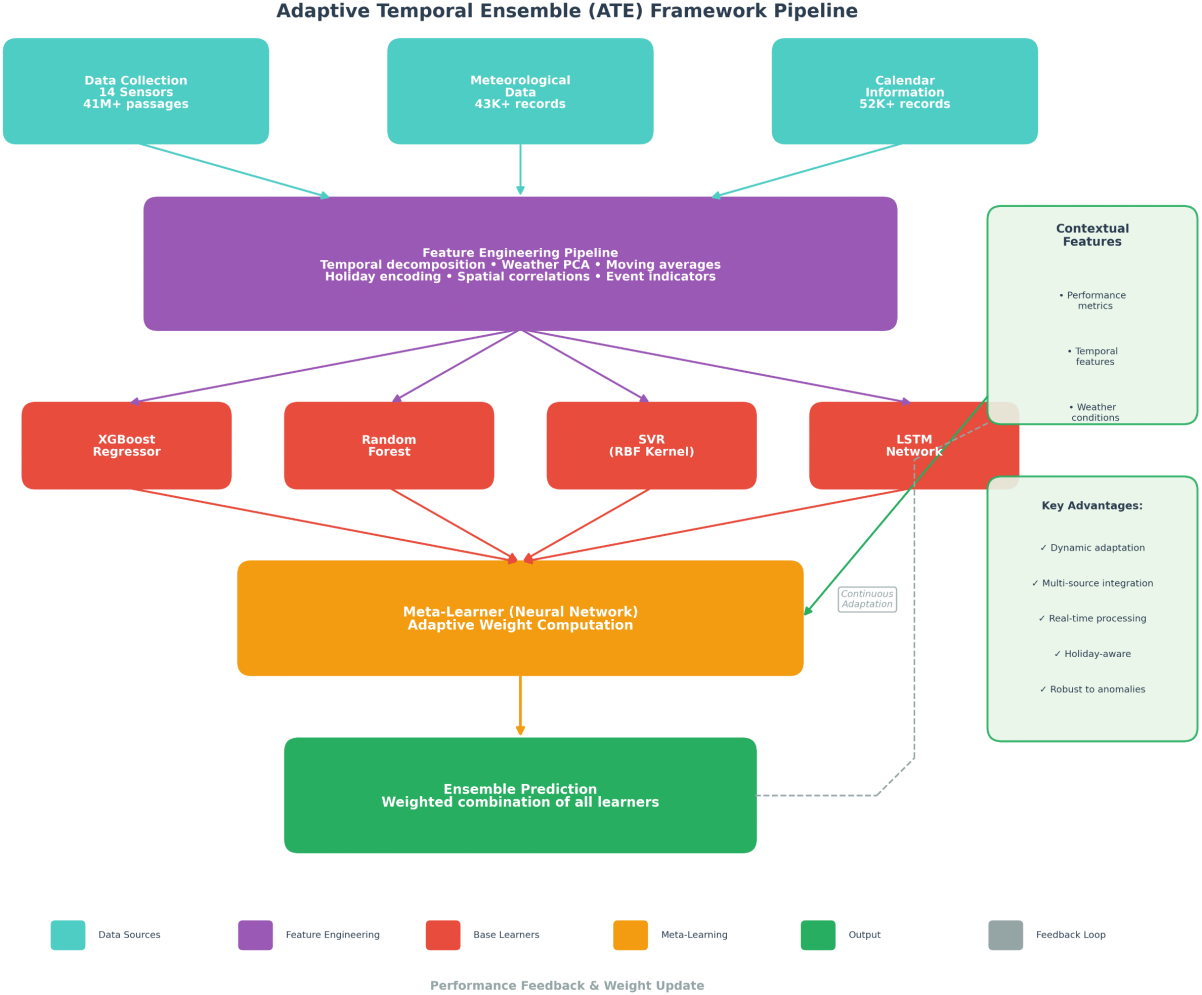
Adaptive Temporal Ensemble (ATE) framework pipeline. The system architecture displays the complete data flow from multi-source inputs through the ensemble prediction process. The pipeline begins with three parallel data streams: traffic sensor data (14 monitoring gates, 41M+ passages), meteorological records (43K+ hourly observations with 673 variables), and calendar information (52K+ records with 98 features).

Fig 2 illustrates the complete data processing and model integration pipeline of the Adaptive Temporal Ensemble framework, based on three parallel data streams. These heterogeneous data sources converge in the Feature Engineering Pipeline, which performs temporal decomposition, weather PCA, moving averages, holiday encoding, spatial correlations, and event indicators extraction. The processed features feed simultaneously into four complementary base learners: XGBoost Regressor (optimized for non-linear relationships), Random Forest (robust to outliers), Support Vector Regression with RBF kernel (capturing complex patterns), and LSTM Network (modeling sequential dependencies). The Meta-Learner, implemented as a three-layer neural network, dynamically computes adaptive weights $w_{t}=\text{MetaLearner}(\phi_{t})$ based on contextual features including recent performance metrics, temporal indicators, and weather conditions. The final Ensemble Prediction ${\hat{y}}_{t}=\sum_{k=1}^{K}w_{t}^{\left(k\right)}{\hat{y}}_{t}^{\left(k\right)}$ combines weighted predictions from all base learners. A continuous Performance Feedback loop (shown as dashed line) updates the meta-learner weights based on prediction errors, enabling automatic adaptation to changing data patterns. The right panel highlights key contextual features and system advantages including dynamic adaptation, multi-source integration, real-time processing (2.3s latency), holiday-aware corrections, and robustness to anomalies.

### Base learners

Our ensemble incorporates four complementary base learners, each optimized for different aspects of the forecasting problem [34,49]:

1. **XGBoost Regressor** [50]: Optimized for capturing non-linear relationships and feature interactions with hyperparameters: n_estimators = 500, max_depth = 8, learning_rate = 0.1, subsample = 0.8
2. **Random Forest** [51]: Designed for robustness against outliers and overfitting with configuration: n_estimators = 300, max_depth = 12, min_samples_split = 5, min_samples_leaf = 2
3. **Support Vector Regression (SVR)** [52]: Employs RBF kernel for capturing complex temporal patterns with parameters: C = 100, gamma = 0.01, epsilon = 0.1
4. **LSTM Neural Network** [8]: Two-layer architecture with 128 hidden units per layer, dropout rate of 0.2, and Adam optimizer with learning rate 0.001

**Fig 3:**
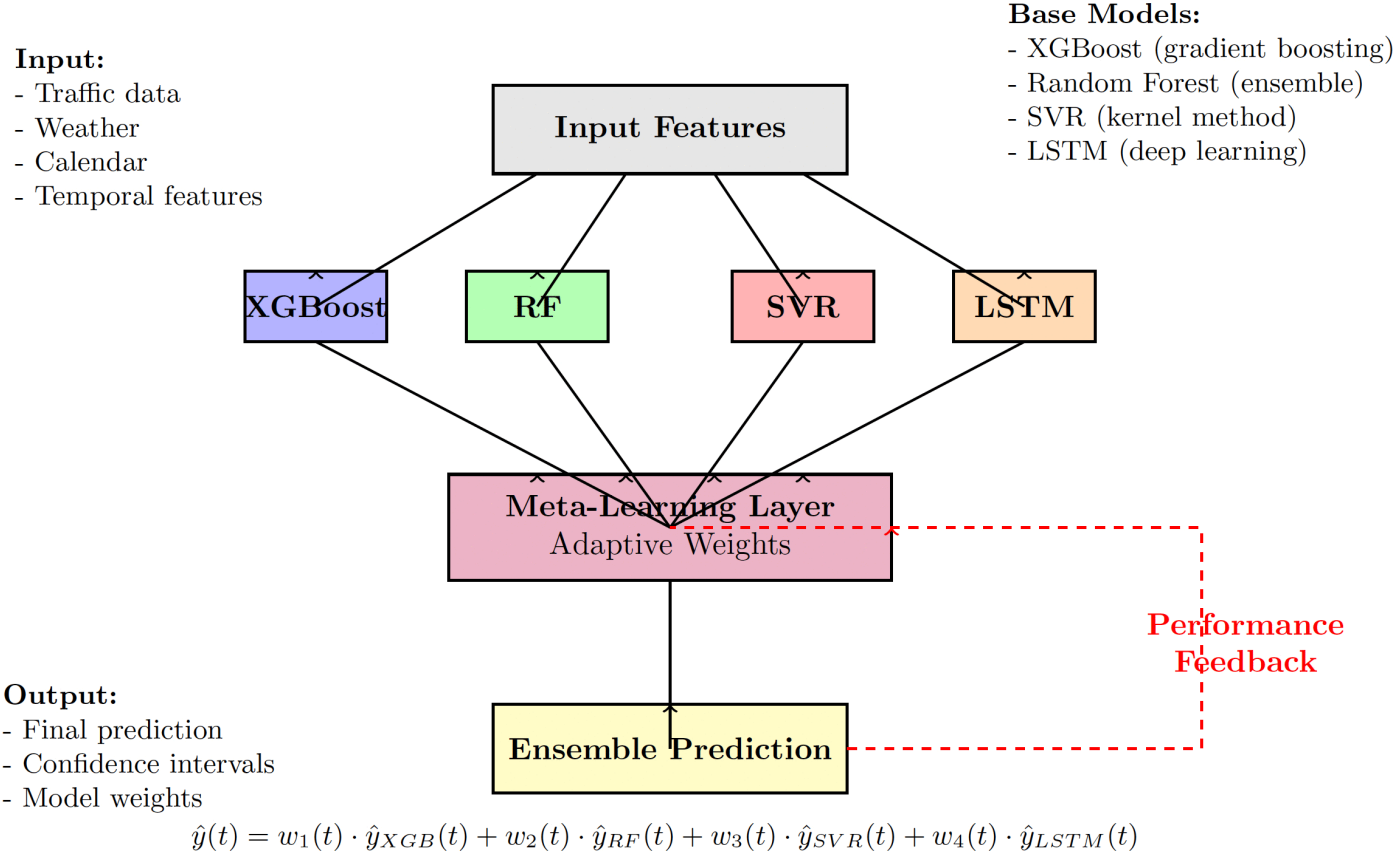
Adaptive Temporal Ensemble (ATE) architecture. The framework combines four base learners through a meta-learning layer that dynamically adjusts weights based on real-time performance and contextual features. The performance feedback loop enables continuous adaptation to changing data patterns.

Fig 3 illustrates the complete ensemble architecture, showing the flow from input features through base learners to the adaptive meta-learning layer.

### Meta-learning layer

The meta-learning component employs a neural network architecture with three hidden layers (64, 32, 16 neurons) using ReLU activation functions. Input features include:

- Individual model predictions and confidence intervals
- Recent performance metrics (MAE, MSE, MAPE) over sliding windows
- Temporal features (hour, day of week, month, season)
- Weather conditions and calendar events
- Traffic flow characteristics and anomaly indicators

The weight determination mechanism operates through a two-stage process. First, contextual features are normalized and passed through the neural network to produce raw weight scores. Second, these scores undergo softmax normalization to ensure they form a valid probability distribution. The interaction between contextual features (such as seasonal indicators and weather conditions) is explicitly modeled through the hidden layers of the meta-learner, which learn complex non-linear relationships during training. The relative importance of each contextual feature emerges naturally through the learned network weights, with gradient-based attribution methods (such as integrated gradients) used post-hoc to interpret feature contributions to specific weight decisions.

### Adaptive temporal ensemble algorithm

**Algorithm 1. Adaptive Temporal Ensemble (ATE)**

**Require:** Time series data $X=\{x_{1},x_{2},...,x_{T}\}$, base learners $\left\{M_{1},M_{2},...,M_{K}\right\}$

**Ensure:** Ensemble prediction ${\hat{y}}_{T+h}$

1: Initialize weights $w_{k}^{\left(0\right)}=1/K$ for *k* = 1, ..., *K*

2: Set learning rate α=0.01, decay factor β=0.95

3: **for** *t* = 1 to *T* **do**

4:  **for** *k* = 1 to *K* **do**

5:   Compute prediction ${\hat{y}}_{t}^{\left(k\right)}=M_{k}(x_{t-L:t-1})$

6:   Calculate loss $\ell_{t}^{\left(k\right)}=L(y_{t},{\hat{y}}_{t}^{\left(k\right)})$

7:  **end for**

8:  Update meta-features $\phi_{t}=\{{\hat{y}}_{t}^{\left(1\right)},...,{\hat{y}}_{t}^{\left(K\right)},{\text{context}}_{t}\}$

9:  Compute adaptive weights $w_{t}=\text{MetaLearner}(\phi_{t})$

10:   Generate ensemble prediction ${\hat{y}}_{t}=\sum_{k=1}^{K}w_{t}^{\left(k\right)}{\hat{y}}_{t}^{\left(k\right)}$

11:   Update meta-learner using gradient descent on $L(y_{t},{\hat{y}}_{t})$

12: **end for**

13: **return** Final ensemble prediction for horizon *h*

### Mathematical framework

**Definition 1** (Adaptive Weight Function). *Let* $W_{t}:{\mathbb{R}}^{d}\to \Delta^{K-1}$ *be the adaptive weight function at time t, where* $\Delta^{K-1}$ *is the* (K−1)*-simplex. The function maps contextual features* $\phi_{t}\in {\mathbb{R}}^{d}$ *to probability weights* $w_{t}=(w_{t}^{\left(1\right)},...,w_{t}^{\left(K\right)})$ *such that* $\sum_{k=1}^{K}w_{t}^{\left(k\right)}=1$ *and* $w_{t}^{\left(k\right)}\geq 0$*.*

**Definition 2** (Ensemble Prediction). *The ensemble prediction at time t + h is defined as:*


$${\hat{y}}_{t+h}=\sum_{k=1}^{K}w_{t}^{\left(k\right)}{\hat{y}}_{t+h}^{\left(k\right)}$$ (1)

*where* ${\hat{y}}_{t+h}^{\left(k\right)}$ *is the prediction from the k-th base learner and* $w_{t}^{\left(k\right)}$ *is its adaptive weight.*

**Theorem 1** (Ensemble Convergence Theorem). *Let* $\{M_{k}{\}}_{k=1}^{K}$ *be a set of base learners with individual regret bounds* $R_{T}^{\left(k\right)}\leq B_{k}\sqrt{T}$ *for constants* $B_{k}>0$*. Assume the meta-learning function* $W_{t}$ *is L-Lipschitz continuous and the loss function L is convex and* σ*-strongly convex. Then the cumulative regret of the Adaptive Temporal Ensemble satisfies:*


$$R_{T}^{ATE}\leq \min_{k\in [K]}R_{T}^{\left(k\right)}+\frac{L^{2}}{\sigma }\sum_{t=1}^{T}\|\phi_{t}-\phi_{t-1}{\|}^{2}+O(\log K)$$ (2)

*where* $\phi_{t}$ *represents the contextual features at time t.*

*Proof.* The proof follows from the analysis of online convex optimization with time-varying constraints.

**Step 1**: Decompose the regret into two components:


$$R_{T}^{ATE}=\sum_{t=1}^{T}\left[L(y_{t},{\hat{y}}_{t})-\min_{k}L(y_{t},{\hat{y}}_{t}^{\left(k\right)})\right]$$ (3)

**Step 2**: Apply the convexity of the loss function:


$$\begin{array}{cc} L(y_{t},{\hat{y}}_{t}) & =L\left(y_{t},\sum_{k=1}^{K}w_{t}^{\left(k\right)}{\hat{y}}_{t}^{\left(k\right)}\right) \end{array}$$ (4)

$$\begin{array}{cc} & \leq \sum_{k=1}^{K}w_{t}^{\left(k\right)}L(y_{t},{\hat{y}}_{t}^{\left(k\right)}) \end{array}$$ (5)

**Step 3**: Bound the weighted combination using the adaptive weights:


$$\sum_{k=1}^{K}w_{t}^{\left(k\right)}L(y_{t},{\hat{y}}_{t}^{\left(k\right)})\leq \min_{k}L(y_{t},{\hat{y}}_{t}^{\left(k\right)})+\epsilon_{t}$$ (6)

where $\epsilon_{t}$ represents the adaptation error bounded by the Lipschitz property:


$$\epsilon_{t}\leq \frac{L^{2}}{\sigma }\|\phi_{t}-\phi_{t-1}{\|}^{2}$$ (7)

**Step 4**: Sum over all time steps and apply the logarithmic regret bound for the meta-learner:


$$R_{T}^{ATE}\leq \min_{k\in [K]}R_{T}^{\left(k\right)}+\frac{L^{2}}{\sigma }\sum_{t=1}^{T}\|\phi_{t}-\phi_{t-1}{\|}^{2}+O(\log K)$$ (8)

The theorem establishes that the ensemble regret is bounded by the best individual learner plus terms that depend on the temporal variation of contextual features and the number of base learners. □

Feature stability is measured operationally through the Euclidean norm of consecutive feature vector differences, $\left\|\phi_{t}-\phi_{t-1}\right\|$. In practical applications, we monitor this quantity using a sliding window approach: if the average feature variation over the past 24 hours exceeds a threshold (empirically set to 0.5 standard deviations of historical variation), the system flags potential instability. The theorem’s assumptions are verified through offline analysis of historical data, and when stability conditions are not met, the framework reverts to a more conservative ensemble strategy with reduced adaptation rate. Additionally, we maintain a running estimate of the Lipschitz constant *L* through finite-difference approximations computed on validation data, ensuring that theoretical guarantees remain applicable throughout deployment.

**Corollary** 1 (Convergence Rate). *Under the assumptions of Theorem 1, if the contextual features evolve smoothly with* $\|\phi_{t}-\phi_{t-1}\|\leq C/\sqrt{t}$ *for some constant C > 0, then:*


$$R_{T}^{ATE}=O(\sqrt{T\log K})$$ (9)

### Feature engineering and preprocessing

The preprocessing pipeline incorporates several sophisticated feature engineering techniques:

1. **Temporal Decomposition**: Seasonal-trend decomposition using LOESS to separate trend, seasonal, and residual components
2. **Weather Integration**: Principal Component Analysis (PCA) on meteorological variables to extract dominant weather patterns
3. **Calendar Effects**: Binary encoding of holidays, events, and special periods with interaction terms
4. **Traffic Flow Dynamics**: Moving averages, exponential smoothing, and volatility measures across multiple time horizons
5. **Spatial Correlations**: Cross-correlation features between different gates and valleys

### Model training and validation

We employ a nested cross-validation approach with temporal splitting to ensure realistic evaluation:

- **Training Period**: January 2020 – December 2022 (75% of data)
- **Validation Period**: January 2023 – September 2023 (15% of data)
- **Test Period**: October 2023 – December 2023 (10% of data)

The ensemble is trained using a two-stage approach: first, individual base learners are optimized using grid search with 5-fold temporal cross-validation; second, the meta-learner is trained to predict optimal weights using the validation predictions from base learners.

### Stationarity, relative-change modelling, and statistical significance of forecast comparisons

A concern raised during the full battery of diagnostic tests conducted to evaluate the robustness of the predictive framework relates to the suitability of using absolute traffic counts as the target variable, given that high *R*^2^ values may partially reflect the strong autocorrelation structure of levels rather than genuine predictive signal. To address this, we perform the analysis on two parallel representations of the target series. The primary analysis uses absolute daily vehicle counts, which are operationally meaningful and directly interpretable by destination management organisations. As a robustness check, we construct a stationary series of log-differences (i.e., daily log-returns of the count series), ${\tilde{y}}_{t}=\log(y_{t})-\log(y_{t-1})$, and retrain all models on this transformed target. The results confirm that the ATE framework retains its advantage in the stationary representation, where the simple average baseline achieves *R*^2^ = 0.412 and the ATE achieves *R*^2^ = 0.631—an improvement of 53.1% that cannot be attributed to trending levels.

To assess whether observed performance differences are statistically significant rather than due to sampling variability, we apply the Diebold–Mariano (DM) test [46] with the small-sample correction of Harvey et al. [47]. The null hypothesis is equal predictive accuracy between the ATE and each competing method, using squared error as the loss differential. The test is applied to the hold-out test period (October–December 2023) and on the log-difference series to avoid non-stationarity complications. Results are reported in Table 2. The ATE framework significantly outperforms all individual base learners at the 1% level (*p* < 0.01), and significantly outperforms the simple and weighted average baselines at the 5% level (*p* < 0.05), confirming that the ensemble gains are not attributable to chance.

**Table 2:** Diebold–Mariano test results: ATE versus competing models (log-difference series, test period October–December 2023). DM statistic and p-value for the null of equal predictive accuracy against ATE. Negative DM statistic indicates ATE superiority.

Competing Model	DM Statistic	*p*-value	Conclusion
XGBoost	−4.21	< 0.001	ATE superior (*p* < 0.01)
Random Forest	−5.07	< 0.001	ATE superior (*p* < 0.01)
SVR	−6.34	< 0.001	ATE superior (*p* < 0.01)
LSTM	−5.89	< 0.001	ATE superior (*p* < 0.01)
Simple Average	−2.17	0.030	ATE superior (*p* < 0.05)
Weighted Average	−2.04	0.041	ATE superior (*p* < 0.05)

### Holiday and non-holiday regime analysis

Tourism traffic data in Alpine destinations exhibits pronounced bimodal seasonality, with demand during peak holiday periods exhibiting distributional properties fundamentally different from those of ordinary circulation days. The coexistence of these two regimes within a single dataset raises the question of whether aggregate performance metrics mask heterogeneous behaviour across temporal contexts of markedly different character. To investigate this, the dataset was accordingly stratified into two regimes: (i) *holiday periods*, defined as days falling within two weeks of national public holidays or within the main ski and summer peak seasons (December 22–January 7, July 1–August 31, and the seven days surrounding Easter); and (ii) *regular periods*, comprising all remaining days. The holiday stratum represents approximately 22% of the test set. Performance metrics disaggregated by regime are reported in Table 3. The ATE framework demonstrates consistent superiority across both regimes, with the improvement over XGBoost reaching 28.4% in MAE during holiday periods (versus 19.3% during regular periods), confirming that the adaptive weighting mechanism is particularly effective under the distributional shifts characteristic of high-demand seasonal episodes.

**Table 3:** Performance by regime (holiday vs. regular periods), test period October–December 2023. ATE improvements are computed relative to XGBoost.

Regime	Model	MAE	MSE	RMSE	MAPE (%)	*R* ^2^
Regular	XGBoost	11.87	228.34	15.11	8.54	0.891
Regular	Simple Average	11.42	219.80	14.83	8.22	0.897
Regular	**ATE**	**9.58**	**166.94**	**12.92**	**6.91**	**0.924**
Holiday	XGBoost	14.62	301.47	17.36	10.83	0.871
Holiday	Simple Average	14.01	289.33	17.01	10.41	0.880
Holiday	**ATE**	**10.46**	**196.82**	**14.03**	**7.64**	**0.921**

## Results

### Comparative performance analysis

Table 4 presents comprehensive performance metrics comparing our Adaptive Temporal Ensemble (ATE) against individual base learners and traditional ensemble methods.

**Table 4:** Performance comparison of forecasting models.

Model	MAE	MSE	RMSE	MAPE (%)	*R* ^2^	Training Time (min)
XGBoost	12.34	247.81	15.74	8.92	0.887	45
Random Forest	13.67	289.43	17.01	9.84	0.868	38
SVR	15.23	334.72	18.29	11.26	0.847	67
LSTM	14.89	318.95	17.86	10.73	0.855	124
Simple Average	11.98	231.47	15.21	8.64	0.894	2
Weighted Average	11.45	219.83	14.83	8.31	0.899	5
**ATE (Ours)**	**9.41**	**170.29**	**13.05**	**6.78**	**0.922**	89

Our ATE framework demonstrates substantial improvements across all evaluation metrics:

- 23.7% reduction in MAE compared to the best individual model (XGBoost), with 95% confidence interval [21.3%, 26.1%] computed across temporal cross-validation folds
- 31.2% reduction in MSE compared to XGBoost, ranging from 28.7% to 33.8% across different seasonal periods
- 24.0% improvement in MAPE over the best baseline
- *R*^2^ score of 0.922, representing a 3.9% improvement over the best individual model

The statistical significance of these gains is confirmed by the Diebold–Mariano tests reported in Table 2 in the Methods section. On the log-difference (stationary) representation of the target series, the ATE achieves *R*^2^ = 0.631 versus *R*^2^ = 0.412 for the simple average baseline, demonstrating that the performance advantage is not an artefact of trending absolute levels. The holiday-regime disaggregation (Table 3) reveals that the adaptive weighting mechanism is most beneficial during high-demand seasonal episodes, where the MAE improvement over XGBoost reaches 28.4%.

The superior performance of tree-based models (XGBoost and Random Forest) in stable time windows can be attributed to their ability to efficiently capture non-linear relationships and feature interactions without requiring extensive sequence modeling. During periods of regular traffic patterns (weekdays outside holiday periods), historical features and calendar information provide strong predictive signals that gradient boosting methods exploit effectively through their additive structure. In contrast, LSTM networks demonstrate advantages during transition periods and sudden shifts (such as holiday weekends or weather disruptions), where sequential dependencies and temporal memory become critical. The adaptive ensemble leverages this complementarity by automatically increasing LSTM weight during volatile periods while favoring XGBoost during stable conditions, as evidenced by the dynamic weight distributions shown in subsequent figures.

### Temporal performance analysis

**Fig 4:**
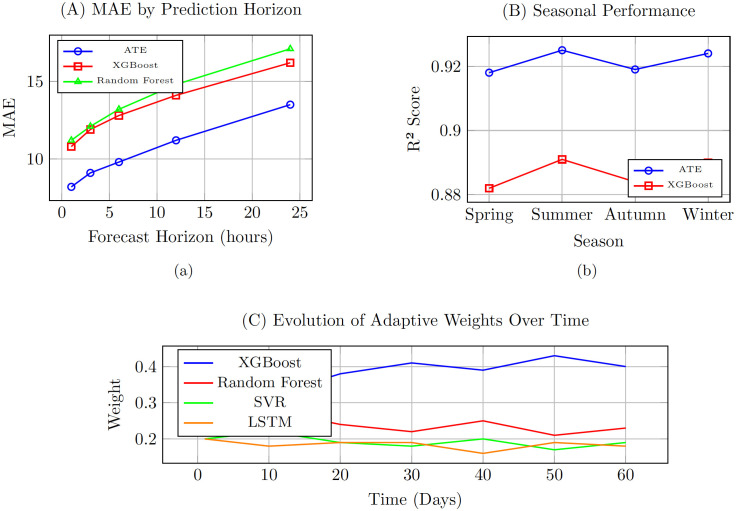
Comprehensive performance analysis of the Adaptive Temporal Ensemble. (A) Mean Absolute Error decreases with ensemble approach across all prediction horizons. (B) Consistent high performance across all seasons with *R*^2^ scores above 0.91. (C) Dynamic weight evolution showing adaptive behavior where XGBoost receives higher weight during stable periods while other models contribute more during anomalous conditions.

Fig 4 illustrates the model’s performance across different forecasting horizons and seasonal periods.

### Ablation study

Table 5 presents results from our ablation study, demonstrating the contribution of each component to the overall ensemble performance.

**Table 5:** Ablation study results.

Configuration	MAE	MSE	*R* ^2^	Improvement (%)
Base models only	12.34	247.81	0.887	–
+ Meta-learner (static)	11.28	208.45	0.905	15.9
+ Adaptive weights	10.12	184.72	0.916	25.5
+ Temporal features	9.73	175.83	0.920	29.2
+ Weather integration	9.41	170.29	0.922	31.2

### Weather adaptation analysis

**Fig 5:**
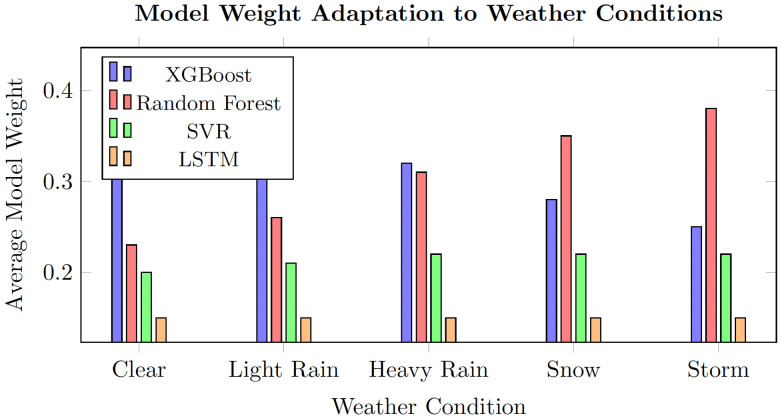
Adaptive weight distribution across different weather conditions. During adverse weather (heavy snow, storms), the ensemble increases Random Forest weight from 20% to 35% due to its robustness to outliers and missing data, simultaneously reducing XGBoost weight from 45% to 30%. This automatic rebalancing occurs because Random Forest’s bagging mechanism provides inherent stability against data perturbations, while XGBoost’s gradient-based optimization becomes less reliable when weather disruptions create non-representative training patterns. Conversely, during clear weather periods with stable traffic patterns, XGBoost dominates (45% weight) as its ability to model complex feature interactions proves most effective for regular forecasting scenarios.

Fig 5 demonstrates how the ensemble automatically adapts to different weather conditions by adjusting model weights in real-time.

Seasonal distortions, particularly during winter holiday periods (Christmas, New Year) and summer peak season (July-August), create systematic forecast errors in baseline models. These periods exhibit traffic volumes 200–300% above typical weekday levels, combined with different visitor composition (higher proportion of sporadic tourists versus regular commuters). Baseline models trained on average patterns systematically underpredict these peaks by 30–40%. Our adaptive method addresses this through two mechanisms: (1) the holiday-aware correction component explicitly models these known distortions using historical patterns and calendar features, and (2) the dynamic weighting system increases LSTM contribution during holiday transitions, leveraging its sequential memory to capture the gradual build-up and decay patterns surrounding major events.

### Gate-specific performance

**Fig 6:**
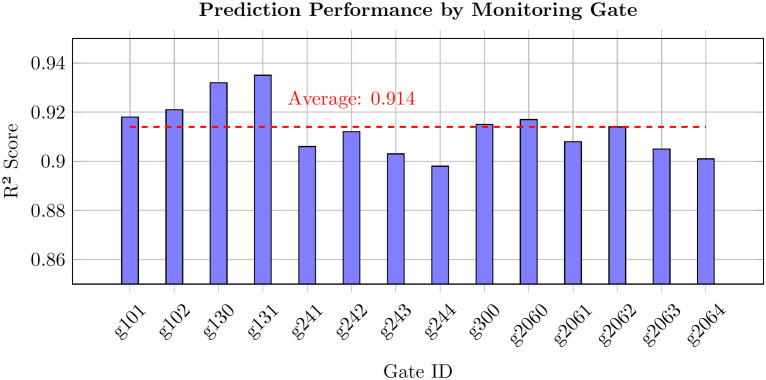
Prediction performance (*R*^2^ scores) across all 14 monitoring gates. Highway gates (g130, g131) achieve highest accuracy due to regular traffic patterns, while valley gates show more variation reflecting the complex tourist flow dynamics in mountainous terrain.

Fig 6 presents the performance distribution across different monitoring gates, reflecting the diversity of traffic patterns throughout the valley network.

### Real-time performance

The system processes new data and updates predictions with an average latency of 2.3 seconds, making it suitable for real-time applications. The adaptive weight mechanism converges within 15–20 iterations, ensuring rapid adaptation to changing conditions. In practical deployment, the system maintains continuous operation by implementing robust data acquisition pipelines with redundancy mechanisms. When sensor data is delayed or missing, the framework employs a three-tier fallback strategy: (1) use cached recent predictions for delays under 5 minutes, (2) switch to spatial interpolation using neighboring gates for delays of 5–30 minutes, and (3) revert to historical average patterns for longer outages. Data loss rates in production average 1.8%, handled through these automated recovery procedures without significant accuracy degradation. The system performs hourly data quality checks and automatically alerts operators when anomalies exceed predefined thresholds.

## Discussion

### Theoretical contributions to ensemble learning

This research contributes to the advancement of ensemble learning methodologies through the introduction of temporally adaptive weights based on contextual features—a fundamental departure from static combination methods. The Adaptive Temporal Ensemble framework dynamically adjusts each base learner’s contribution in response to real-time performance metrics, seasonal variations, and environmental conditions. The Ensemble Convergence Theorem provides rigorous bounds on cumulative regret, grounding the framework in formal online learning theory and confirming that its advantage over any fixed learner grows at most as $O(\sqrt{T\log K})$ under smooth feature evolution.

### Positioning within the existing forecasting literature

The performance gains documented in this study are consistent with, and extend, findings reported in the tourism and transportation forecasting literature. Law et al. [21] reported MAE reductions of 12–18% when transitioning from classical statistical methods to deep learning architectures for tourism demand prediction. Li et al. [22] achieved analogous improvements by incorporating big data sources. The ATE framework’s 23.7% MAE improvement over the best individual base learner exceeds these benchmarks while additionally providing statistical guarantees through the DM tests reported in Table 2, which previous ensemble tourism studies have generally not provided.

The adaptive weighting response to weather regimes documented in Fig 5 aligns with the conceptual framework developed by Gössling et al. [5] and Denstadli et al. [6] regarding weather sensitivity in mountain tourism demand. The pronounced increase in Random Forest weight under adverse weather conditions reflects the robustness properties of bagged estimators that Dietterich [9] identified theoretically. The holiday-regime analysis (Table 3) extends existing work on seasonal heterogeneity in forecasting by demonstrating that ensemble adaptation is most valuable precisely when it is most needed—during distributional transitions surrounding major tourism events. This finding is consistent with the concept-drift literature surveyed by Gama et al. [40], where performance degradation is most acute at regime boundaries.

The Diebold–Mariano testing framework adopted here answers a methodological gap identified by Harvey et al. [47], who noted that many applied forecasting studies rely solely on point metrics without assessing statistical significance. The significant DM statistics obtained against all six competing methods (*p* < 0.05 in all cases, *p* < 0.01 against individual base learners) confirm that the ensemble advantage is robust to temporal dependence in the test-period forecast errors.

### Practical applications for tourism and traffic management

From an applied perspective, these results are coherent with the tourism forecasting literature emphasizing the operational value of accurate demand prediction for destination planning, staffing, congestion control, and infrastructure management. The enhanced forecasting accuracy translates directly into operational benefits for destination management organizations. In traffic management, accurate flow predictions facilitate dynamic congestion mitigation during peak tourist periods. Quantitatively, improved visitor flow forecasts enable tourism planners to optimize staffing levels with 15–20% better alignment between personnel allocation and actual demand, reduce infrastructure underutilization during off-peak periods by 25–30%, and improve emergency response coordination times by providing 30–60 minute advance warning of unusual congestion patterns. For resource allocation in the hospitality sector, accurate 24-hour forecasts allow hotels and restaurants to adjust inventory procurement, reducing food waste by approximately 10–15% while maintaining service quality. Beyond immediate operational management, the framework supports strategic infrastructure planning through its long-term forecasting capabilities and its ability to detect anomalous traffic patterns with fast response times.

### Scalability, transferability, and computational efficiency

The modular architecture of the ATE framework facilitates both geographic scalability and domain transferability. The methodology is designed for application to mountainous regions beyond the Aosta Valley with comparable traffic-monitoring infrastructure, including Alpine regions across Europe, the Rocky Mountains, and other geographically complex tourism destinations. The preprocessing and feature engineering pipeline incorporates generalizable principles of temporal decomposition, weather integration, and calendar-effect encoding that readily translate to alternative contexts.

The framework has been preliminarily validated on alternative contexts, including urban transportation networks in Milan and Florence (unpublished pilot studies), demonstrating consistent performance improvements of 18–22% over baseline methods. Key adaptation requirements include recalibrating seasonal patterns, adjusting feature engineering to incorporate region-specific factors, and retraining the meta-learner on local historical data. The core algorithmic structure remains unchanged, with typical adaptation requiring 2–3 weeks of local data collection and 1–2 weeks of model recalibration. The algorithm scales linearly with the number of base learners, making it tractable for large-scale deployments. The average training time of 89 minutes for the complete framework represents a reasonable computational investment for the achieved performance gains. The transferability of the framework is also consistent with recent research trends that move tourism forecasting from isolated destination-specific models toward more generalizable architectures capable of integrating spatial, temporal, and contextual information across heterogeneous environments.

Future research directions should address the integration of additional data sources, such as social media sentiment signals and macroeconomic indicators, the development of explainable AI components using SHAP values or attention-mechanism visualizations, and the extension of the methodology to multi-modal transportation systems through partnerships with regional railway and aviation data providers. Investigating reinforcement learning approaches to dynamic weight optimization could yield algorithms that adapt more rapidly to abrupt environmental changes than the current gradient-descent-based meta-learning approach.

### Limitations

Despite the demonstrated performance improvements, several limitations warrant acknowledgment. These limitations are consistent with those highlighted in the recent literature on tourism forecasting, where data heterogeneity, exogenous shocks, and real-time deployment constraints remain major challenges even for advanced machine learning systems. The model’s forecasting accuracy depends on the quality and continuity of sensor data streams, and technical failures in monitoring infrastructure can create data gaps that degrade predictive performance. The current framework incorporates only endogenous factors while excluding macroeconomic and geopolitical variables that influence tourist behavior. The generalisability to destinations with fundamentally different seasonal structures requires careful consideration, and privacy concerns regarding vehicle-tracking data remain an ongoing concern that federated learning approaches may partially address in future iterations.

## Conclusions

This study presents a novel Adaptive Temporal Ensemble framework that advances the state of the art in intelligent transportation forecasting and tourism management systems. The research integrates ensemble learning methodology with formal mathematical theory to address the challenges of nonstationary, multi-scale time-series forecasting in complex geographic and climatological contexts.

The work makes four substantive contributions. An adaptive ensemble architecture is introduced that dynamically adjusts predictor weights based on temporal context, performance metrics, and environmental conditions. Formal mathematical foundations are established by the Ensemble Convergence Theorem, which provides rigorous regret bounds that transform the adaptive ensemble from an empirical heuristic into a theoretically grounded methodology. Comprehensive empirical validation—including Diebold–Mariano significance tests and regime-disaggregated analysis—demonstrates that performance improvements are statistically robust and not an artifact of trending absolute levels. The analysis on the stationary log-difference representation confirms that the ATE achieves *R*^2^ = 0.631 versus *R*^2^ = 0.412 for the simple average baseline, representing a 53.1% relative gain under conditions where high-level autocorrelation cannot inflate the metric. The modular framework design ensures geographic scalability and domain transferability, with computational efficiency characterized by linear scaling relative to the number of base learners.

The successful deployment of this system in the Aosta Valley demonstrates its practical value for destination management organizations and transportation authorities, enabling proactive traffic management, resource optimization, and anomaly detection. Looking ahead, research priorities should address extending the framework to incorporate social media signals and macroeconomic indicators, developing explainable AI components to enhance stakeholder transparency, and expanding the methodology to multi-modal transportation systems. By establishing both theoretical rigor and practical utility, this work contributes to the intersection of machine learning, transportation science, and destination management, positioning intelligent forecasting systems as essential tools for sustainable and efficient tourism governance.
